# Two Cases of Azoturia

**Published:** 1885-04

**Authors:** J. Wallace Marsh

**Affiliations:** Schoharie, New York


### II.—TWO CASES OF AZOTURIA.

BY J. WALLACE MARSH, D.V.S.

1*st Case.*—I was called to see a bay mare of very fine conformation, aged six, owned by a farmer and worked as a farm horse usually is. She had stood in the stable for some four or five days prior to the attack, and when taken out she was to all appearances in prime condition of health and spirits. She was harnessed and driven about two miles, when she began to hump or draw up the muscles of the loins; broke out in a profuse perspiration—body trembling violently: was with great difficulty removed to a neighboring stable, where she broke down on the fetlocks behind; went down on the floor three times—managed to raise herself twice, the third time was unable to regain her feet, when she lost all control of the posterior parts. This was the condition I found her in. Entire body terribly convulsed. I proceeded at once to empty the bladder by the use of the catheter. Found the urine to be of a very dark or coffee-color, very thick, consistency of linseed oil or syrup. Specific gravity about 1035. Pulse, 70. Temperature, 103. Muscles of the back and posterior parts rigid and tense. From the history of the case and the symptoms presented I diagnosed this to be a case of azoturia. I at once administered 4 grs. of morphia hypodermically; ordered her to be well covered; rugs dipped in hot water applied to the back; gave a cathartic, also diaphoretic, together with diuretics. Used enemas of warm water. Would say there was great tympanitis of the bowels in this case.

2d Day.—Urine voided readily, and of considerable quantity. Continued treatment.

3d Day.—Applied mustard blister to the back. Other treatment same.

4th Day.—No action of the purge. Gave another cathartic ball, which had no effect. Gave no more, but used enemas. Bowels began to act as normal. Tympanitis reduced. Then commenced to give nux vomica.

5th Day.—Mare was able to get on her feet, but was unable to stand. Had her put in slings, when she gradually began to gain some control of her hind parts, and seemed to improve for about eight days, when, all at once, she began to weaken behind, and her legs to swell. Indications of a cerebral nature presented themselves, and the mare died three days after. I had her fed on wheat bran mashes, and gave plenty of water to drink.

*2d Case.*—A bay horse, aged 6 years, taken in exactly the same way, only in a still more violent manner. Treatment similar. Gave chloral hyd. to ease and quiet animal. Had to give two doses of aloes to get action of the bowels, when I obtained a purging action, which lasted for about eighteen hours. After this, horse seemed to improve a little, but died on the fourth day, not having any action of the posterior parts.

These cases puzzled me very much, indeed. I perused the report of Prof. Porter, in the JOURNAL, on them, but must say I have my doubts as to the prognosis. In these violent cases will you please advise me. If you can publish these rough descriptions, do so, as it may bring out some other opinions.

Schoharie, New York.

